# Association between periorbital cutaneous leishmaniasis and basosquamous carcinoma

**DOI:** 10.1590/0037-8682-0758-2020

**Published:** 2021-03-08

**Authors:** Danielle Santos Grisolia, Ricardo Vieira Teles-Filho, Adriana Oliveira Guilarde

**Affiliations:** 1 Secretaria de Saúde do Distrito Federal, Brasília, DF, Brasil.; 2 Universidade Federal de Goiás, Faculdade de Medicina, Goiânia, GO, Brasil.; 3 Universidade Federal de Goiás, Instituto de Patologia Tropical e Saúde Pública, Goiânia, GO, Brasil.

**FIGURE 1:**
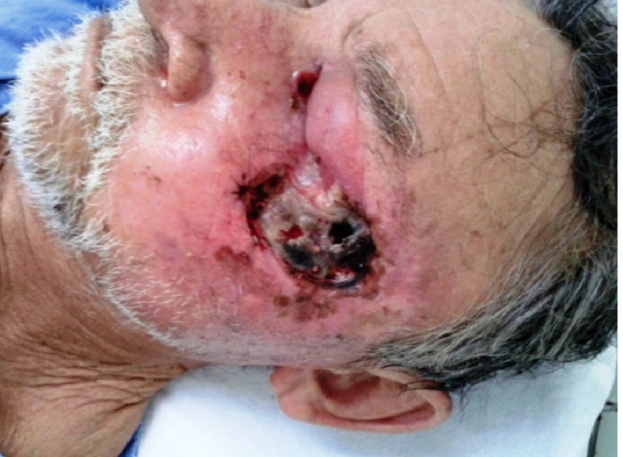
Ulcerated lesion with necrosis and bone exposure.

**FIGURE 2:**
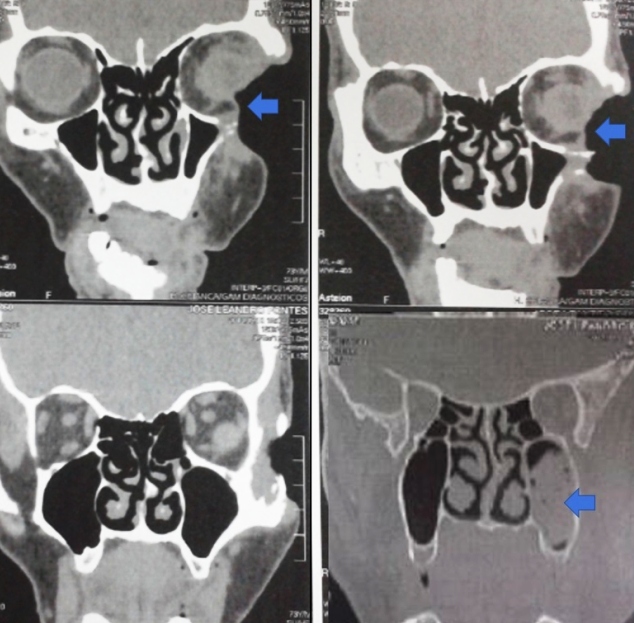
Destruction of the left orbit and left zygomatic bone. Left maxillary sinus filling (skull computed tomography).

A 73-year-old male rural worker from the Brazilian Amazon region presented with a 4-year left facial ulceration, which became painful as it increased in size. Physical examination revealed a deep 8-cm ulcer in the left malar and periorbital regions, associated with necrosis and purulent exudation (Figure 1). Skull computed tomography showed an osteolytic and infiltrative lesion in the malar and frontal regions (Figure 2).

**FIGURE 3:**
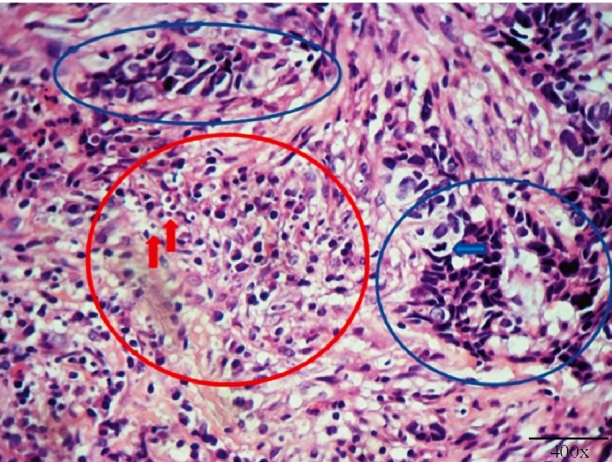
Image of skin sample showing basaloid cells with scarce cytoplasm and hyperchromatic, irregular nuclei (blue circles); mitotic figure (blue arrow); lympho-histio-plasmocitary inflammatory infiltrate (red circle); vacuoles with ovoid corpuscles within histiocytes (red arrows) (haematoxylin & eosin staining; 400x magnification).

Histopathological examination of the lesion revealed atypical basaloid and squamous cell proliferation, with ovoid microstructures within the histiocyte cytoplasm, suggestive of leishmaniasis (Figure 3). Polymerase chain reaction identified *Leishmania viannia*. Immunohistochemical analysis of the samples revealed P-63-positivity, whereas the epithelial membrane was antigen-negative, confirming the diagnosis of basosquamous carcinoma (BSC).

Treatment with meglumine antimoniate for tegumentary leishmaniasis (TL) produced a satisfactory clinical response. The patient underwent radiotherapy and enucleation and was scheduled for reconstruction.

To our knowledge, this is the first case describing the association between BSC and TL. TL may impair the immune system, thereby reducing clonal cell susceptibility to destruction^1^. This case highlights leishmaniasis as a differential diagnosis of complicated mucocutaneous lesions^2^, thus establishing a new reference for future studies investigating the interaction between infectious and neoplastic diseases, and it may even strengthen recent hypotheses of a direct correlation between leishmaniasis and skin carcinomas^3^.
